# Carbon quantum Dot incorporated Xanthan gum based gel polymer electrolytes for high performance supercapacitors

**DOI:** 10.1038/s41598-025-02341-z

**Published:** 2025-05-25

**Authors:** Manisha Sudhakar Alva, Ronald Aquin Nazareth, Y. N. Sudhakar, Nakul Desai

**Affiliations:** 1 Department of Chemistry, St Aloysius (Deemed to be University), Mangaluru, 575003 Karnataka India; 2 Department of Chemistry, Mangalore University, Mangalagangotri, Mangaluru, 574199 Karnataka India; 3 Department of Chemistry, Manipal Institute of Technology, Manipal Academy of Higher Education, Manipal, 576104 India

**Keywords:** Gel polymer electrolyte (GPE), Carbon quantum Dot, Xanthan gum, Electrochemical performance, Supercapacitor, Energy storage, Supercapacitors

## Abstract

In this study, a novel biodegradable gel polymer electrolyte (GPE) was developed using carbon quantum dots (CQDs)-infused xanthan gum (XG) as the polymer matrix, sodium perchlorate (NaClO_4_) as the ionic dopant, and glycerol as the plasticizer. The GPE was paired with activated carbon (AC) and graphene (GC) electrodes to fabricate symmetric supercapacitor cells to enhance energy storage performance. Xanthan gum underwent hydrothermal treatment to form a distinctive puffer ball-like microstructure, which was further nucleated into CQDs. This study introduced an innovative approach by incorporating carbon quantum dots into a polymer electrolyte system, with a new focus on investigating the interactions between the polymer matrix and the salt, offering new insights into their integrated performance. These CQDs functioned as stabilizers and enhanced both the ionic conductivity and electrochemical behavior of the GPE. Structural and morphological analyses, including X-ray diffraction (XRD), scanning electron microscopy (SEM), and transmission electron microscopy (TEM), confirmed a wave-like, porous surface and well-dispersed CQDs. Fourier transform infrared spectroscopy (FTIR) and thermogravimetric analysis (TGA) revealed strong intermolecular interactions among the GPE constituents, indicating excellent thermal and chemical stability. Electrochemical studies showed that the AC electrode achieved a specific capacitance of 92 F g⁻¹ via cyclic voltammetry (CV), while the GC electrode delivered 69 F g⁻¹. Galvanostatic charge-discharge (GCD) tests at 1 mA g⁻¹ showed that the GC electrode reached specific capacitance of 75 F g⁻¹, with energy density and power density of 10.40 Wh kg⁻¹ and 0.49 kW kg⁻¹ respectively. Similarly, AC electrode-based supercapacitor was tested which showed specific capacitance, energy density and power density as 45 F g⁻¹, 5.55 Wh kg⁻¹, and 0.66 kW kg⁻¹, respectively. Both systems demonstrated good reversibility and cycling stability, highlighting the potential of CQD-integrated biodegradable GPEs and carbon-based electrodes for environmentally friendly, flexible, and high-performance supercapacitor applications.

**Supplementary Information:**

The online version contains supplementary material available at 10.1038/s41598-025-02341-z.

## Introduction

The growing global demand for efficient, reliable, and sustainable energy storage solutions has spurred significant interest in advanced technologies. As the world transitions to renewable energy sources and electric mobility, the need for high-performance energy storage devices to bridge the gap between conventional batteries and capacitors becomes increasingly critical. Research into supercapacitors is essential for addressing these challenges, enabling breakthroughs in sustainable energy applications, and contributing to a greener and more energy-efficient future. Supercapacitors are promising energy storage technologies that have gained significant attention from the scientific community because of their low cost, high power density, long cycle life, and exceptional cycling stability. They work well as temporary energy storage devices in energy harvesting systems, which gather energy from renewable or ambient sources such as light, mechanical motion, or electromagnetic fields and transform it into electrical energy in an energy storage device^1–5^. Despite significant progress, challenges such as high costs, complex preparation processes, environmental concerns, and leakage hinder their practical applications. Consequently, advancing electrode fabrication technologies and exploring novel electrolyte systems are crucial for developing high-performance supercapacitors^6^.

Supercapacitors are classified into Electric Double-Layer Capacitors (EDLCs), Pseudocapacitors, and Hybrid Supercapacitors based on their charge storage mechanisms. EDLCs utilize high-surface-area materials like activated carbon, Carbon Nanotubes, and graphene for electrostatic charge storage, while pseudocapacitors depend on fast, reversible Faradaic reactions involving MnO₂, NiO, and conducting polymers. Functionalized nanomaterials enhance their electrochemical and mechanical properties^7^. Hybrid supercapacitors integrate both mechanisms, often using asymmetric configurations with carbon materials, polymers, and metal oxides to improve energy and power densities^8^. Supercapacitor performance depends heavily on electrode and electrolyte materials; biomass-derived activated carbons and graphene offer high surface area and conductivity^9^, while redox-active materials like Co(OH)₂, Ni(OH)₂, and MnO₂^10–15^ contribute to enhanced capacitance. To address the limitations of liquid electrolytes, gel polymer electrolytes (GPEs) have gained attention for their safety, stability, and compatibility. Incorporating biodegradable biopolymers into GPEs further supports the development of eco-friendly supercapacitors^16,17^.

Gel polymer electrolytes (GPEs) have gained prominence as a promising solution for next-generation energy storage, particularly in all-solid-state supercapacitors, due to their superior ionic conductivity and enhanced electrode–electrolyte interfacial contact compared to solid polymer electrolytes^18,19^. Their hybrid structure comprising a polymer matrix, plasticizer, and conducting salt enables faster ion transport and improved electrochemical performance. These properties can be tailored through strategies such as ionic doping, polymer blending, and incorporation of nanomaterials like graphene, carbon nanotubes (CNTs), and carbon dots. Recent studies have demonstrated the positive impact of nanofillers and ionic liquids on ionic mobility, electrochemical stability, and cycling life^20–22^. In parallel, biodegradable and naturally derived polymers such as agarose, cellulose, and xanthan gum are being explored as sustainable GPE matrices due to their environmental compatibility, mechanical robustness, and intrinsic ionic conductivity^23,24^. Unlike liquid electrolytes, which are prone to leakage and poor thermal stability, GPEs immobilize the electrolyte within a polymer network, enhancing safety, mechanical integrity, and longevity, thereby making them ideal for flexible and wearable supercapacitor devices^25,26^.

In recent years, increasing environmental concerns and the push for sustainable energy have driven interest in biodegradable materials for energy storage. Conventional synthetic polymers like Poly(vinylidene fluoride) (PVDF) and Polytetrafluoroethylene (PTFE) are costly, solvent-dependent, and environmentally unfriendly. In contrast, natural biopolymers such as xanthan gum (XG), cellulose, and chitosan offer advantages like abundance, renewability, and non-toxicity^27^. XG, a microbial polysaccharide from Xanthomonas campestris, features a unique structure with high viscosity and gelation ability, even at low temperatures, making it ideal for GPE applications^28^. XG-based GPEs have shown strong electrochemical performance, with specific capacitance up to 347 F g⁻¹ and energy density of 24 µWh cm⁻², along with high ionic conductivity and stability, even in the presence of aqueous sulfate ions^29^. XG GPE achieved a specific capacitance which notably exceeds the 51.1 F g⁻¹ reported for chitosan-based macromolecule cross-linked GPEs. Furthermore, the energy density attained in our system is higher than values reported for similar biodegradable systems, such as chitosan-based electrolytes (7.1 Wh kg⁻¹)^25^ and starch-based counterparts (∼5–8 Wh kg⁻¹).

Although many researchers have obtained biodegradable ionic conducting membranes by modifying natural polymers, pectin-based hydrogel electrolytes have attracted attention for their eco-friendliness and ability to form stable ionic networks^30^. Similarly, iota-carrageenan and acacia gum systems have demonstrated good mechanical strength and salt compatibility, enhancing their suitability for flexible energy devices. Polycaprolactone-based GPEs further contribute by offering thermal stability and biodegradability, making them viable for environmentally friendly energy storage applications^31,32^. By chemically or physically altering natural polymers or their derivatives, such as alginate^33^, polyaniline^34^, lignin^35^, and gelatin^36,37^, researchers have produced biodegradable ionic conducting membranes. However, these materials may still face challenges in achieving optimal ionic conductivity and electrochemical performance.

To overcome these limitations, recent investigations have focused on incorporating nanomaterials such as carbon quantum dots (CQDs) to enhance ion transport and provide additional pseudocapacitive behavior. CQDs, owing to their high surface area, tunable surface functionalities, and superior dispersibility in polymer matrices, have emerged as multifunctional additives in energy storage systems^38^. The Mn_2_O_3_/CQD_2_ electrode developed by Kiey et al.^39^ exhibited a high specific capacitance of 612 F g^− 1^ at 1 A g^− 1^ in a three-electrode arrangement. However, most reported CQD-incorporated systems employ synthetic polymers or ionic liquids, which compromise biodegradability. Moreover, combinations of CQDs with natural biopolymers like xanthan gum remain scarce^40^. Although xanthan gum-based films plasticized with glycerol^41–43^, and salt-doped biopolymer systems^32,44–46^have shown promise individually, a comprehensive, biodegradable GPE system integrating xanthan gum, glycerol, sodium perchlorate, and carbon quantum dots has not yet been reported for supercapacitor applications highlighting a novel and sustainable direction for future research.

In this work, we report the fabrication and electrochemical evaluation of a novel, biodegradable gel polymer electrolyte (GPE) designed for supercapacitor applications. The GPE was prepared using xanthan gum as the biopolymer matrix, glycerol as a plasticizer, sodium perchlorate (NaClO₄) as the ionic salt, and carbon quantum dots (CQDs) as a conductive nanofiller. This unique combination was chosen to synergistically enhance ionic conductivity, mechanical flexibility, and electrochemical performance while maintaining environmental compatibility. Two types of carbon-based electrodes activated carbon (AC) and graphene (GC) were used to construct symmetric supercapacitor configurations, allowing comparative insight into electrode–electrolyte interactions. To the best of our knowledge, this is the first report demonstrating the incorporation of CQDs into a xanthan gum-based GPE doped with NaClO₄ for use in supercapacitors. The performance of the supercapacitor was evaluated via AC impedance spectroscopy, cyclic voltammetry (CV), and galvanostatic charge-discharge (GCD) techniques, which demonstrated the effectiveness and potential of the GPE system for high-performance energy storage applications.

## Experimental

### Materials

Xanthan gum (XG) (high molecular weight) was purchased from Sigma–Aldrich and glycerol was purchased from Merck. Sodium perchlorate (Merck) was dried at 393 K and kept under vacuum for 48 h before use. Polyyvinylidene fluoride (PVDF) purchased from BLD pharmatech, n-Methyl-2-pyrrolidone (NMP) purchased from SRL chemicals, activated carbon was obtained from areca fibers with a surface area of 250 m^2^ g^− 1^. Hydrazine hydrate and graphene powder purchased from Merck. Stainless steel of thickness 0.15 mm was used as a substrate.

### Preparation of carbon quantum Dots (CQDs)

Carbon quantum dot (CQD) solutions were synthesized from xanthan gum. In a standard CQD synthesis procedure, a mixture of 0.1 g of xanthan gum polymer and 1 ml of hydrazine hydrate was ultrasonically treated in 10 ml of water for 30 min. This solution was then put into a 25 ml stainless autoclave lined with Teflon, sealed, and heated for an additional 10 h at 150 °C in an electric oven. Followed by cooling to room temperature, the resulting product containing CQDs was filtered through Whatman filter paper to eliminate insoluble carbon residues. The purified CQDs were then centrifuged and subsequently subjected to structural characterization and property evaluation.

### Preparation of a biodegradable gel polymer electrolyte (GPE)

A stock solution was prepared by dissolving 3 g of xanthan gum in 250 ml of distilled water. Gel polymer electrolytes (GPEs) were formulated by blending appropriate amounts of salt and plasticizer. For the samples designated S1-S4, 90 wt% of the stock solution was mixed with 10 wt% glycerol (an optimized concentration displaying superior plasticizer retention properties), 1 ml of CQD solution, and varying quantities of NaClO_4_ salt (0.01 g, 0.02 g, and 0.03 g, respectively) (Table 1). These prepared solutions were individually placed in clean 10 ml Petri dishes, allowed to gelate initially at room temperature, and subsequently incubated in an oven at 333 K for 48 h to form GPEs before subsequent analysis.

**Table 1:** Sample label and Wt% of each constituent of the gel polymer electrolyte.

Sample label	CQD (ml)	Dopant (NaClO_4_) (g)
Pure XG	–	–
S1	1	–
S2	1	0.01
S3	1	0.02
S4	1	0.03

### Characterization

Fourier transform infrared spectroscopy (FTIR) analyses of the gel polymer samples, both with and without NaClO_4_ doping were conducted at ambient temperature using a Perkin Elmer Spectrum Two FTIR spectrometer. Thermal gravimetric analysis (TGA) of the undoped and NaClO_4_-doped XG samples was performed with a PerkinElmer TGA 4000 instrument. The measurements involved a temperature range of 300–700 K with a heating rate of 10 °C min^− 1^ under a nitrogen atmosphere at a flow rate of 50 ml min^− 1^, with data acquired from the initial heating cycle. Microscopic imaging of the gel polymer electrolyte (GPE) samples was accomplished by scanning electron microscopy (SEM), specifically with a ZEISS EVO18 special edition. Transmission electron microscopy (TEM) analysis was carried out via an FEI Tecnai G2-30 electron microscope operating at 300 kV. X-ray powder diffraction (XRD) studies were conducted with a Rigaku D/max-2500 instrument equipped with Cu Kα radiation.

### Fabrication of symmetrical cells

**Fig. 1:**
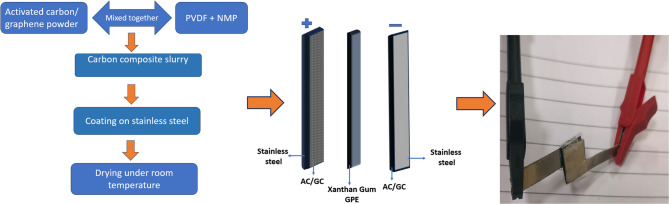
Flow chart depicting the steps involved in the preparation of electrode and fabrication of GPE.

Activated carbon (AC) and graphene powder were used as the electrode materials for the construction of supercapacitors. Carbon composite electrodes were prepared by blending polyvinylidene difluoride (PVDF) and activated carbon (AC)/ graphene powder (GC) in N-methyl-2-pyrrolidone (NMP)^47^. The materials were thoroughly mixed in NMP to form a homogeneous slurry. The slurry was then cast onto a stainless steel used as the substrate and allowed to dry thoroughly overnight. Two supercapacitors were constructed: one with two stainless steel electrodes coated with graphene powder and the second with AC using xanthan gum GPE, i.e., (GC/XG/GC)/(AC/XG/AC) as shown in Fig. 1. The GPE was used to build two supercapacitor cells sandwiched between two produced GC and AC electrodes. The two wires were kept outside the unit cell, which was sealed inside an aluminum pouch coated with plastic. Galvanostatic charge-discharge (GCD), electrochemical impedance spectroscopy (EIS), and cyclic voltammetry (CV) experiments were used to carry out the electrochemical characterization. The electrochemical investigation was conducted with a BioLogic SP-50e device.

## Results and discussion

### FTIR studies

**Fig. 2:**
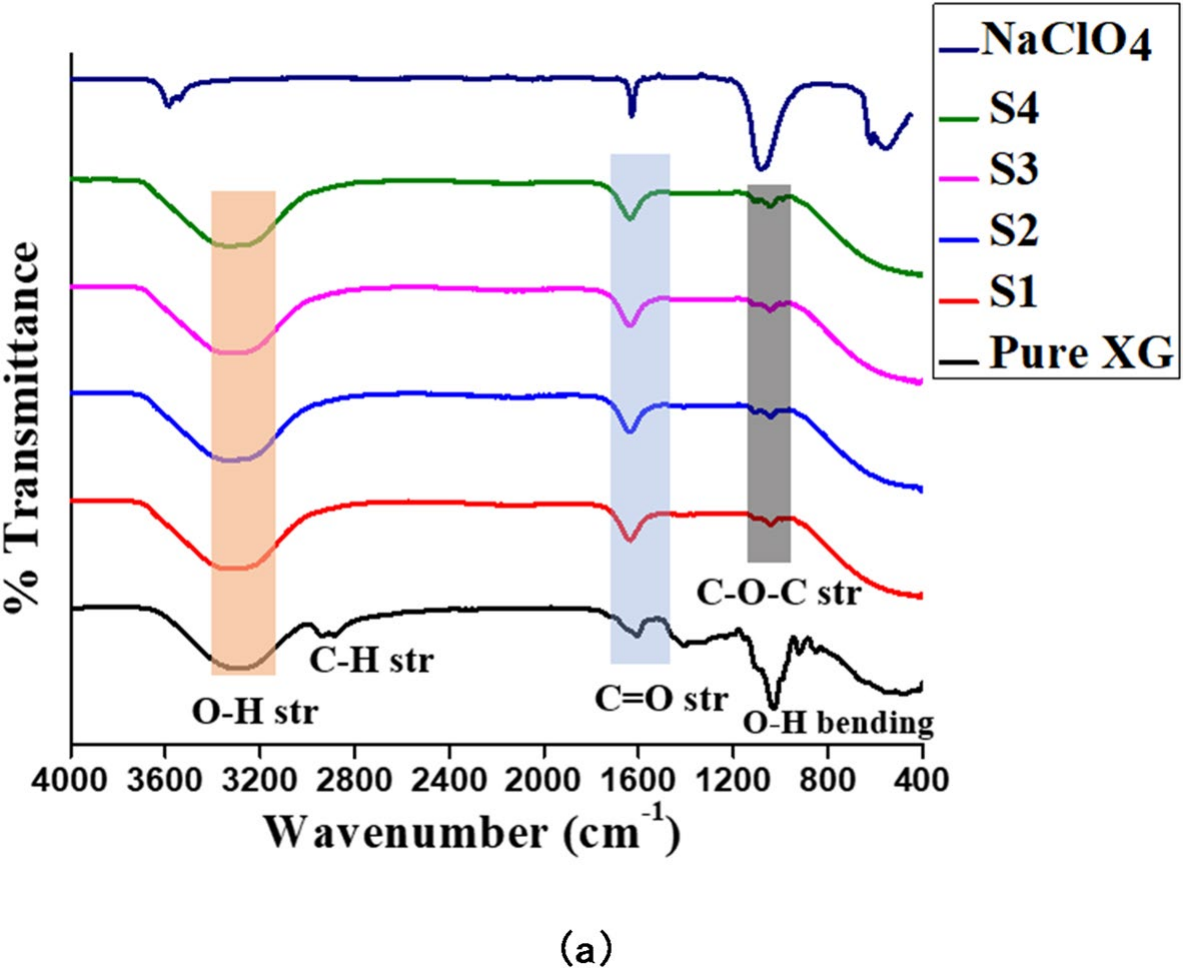
(**a**) FTIR spectra of different gel polymer electrolyte samples and NaClO_4_ salt.

In the Fourier transform infrared (FTIR) spectra (Fig. 2), pure XG exhibited a prominent broad peak at 3284 cm^− 1^ attributed to O-H stretching. Upon the addition of CQDs and sodium salt in samples S1, S2, S3, and S4, this peak shifts to 3339 cm^− 1^, demonstrating the physical interaction between the salts of sodium, CQDs, and the gel polymer. Additionally, significant peaks at 1030 cm^− 1^ and 1044 cm^− 1^, related to O-H bending and C-O-C stretching from glycosidic bonds, respectively exhibit broadening, indicating the occurrence of functional group interactions upon glycerol addition. For the pure XG sample, the C–H stretching vibration was observed as a peak around 2900 cm⁻¹; however, in samples S1, S2, S3, and S4, this C–H stretching peak disappears following the incorporation of CQDs and salt. The absorption band at 1634 cm^− 1^ corresponds to C = O stretching^48–51^. The peak of NaClO_4_ at 618 cm^− 1^ is absent, suggesting its involvement in interactions within the polymer electrolyte system. The observed peak shifting and disappearance within the polymer electrolyte system implies the presence of interactions among XG, salt, and plasticizer.

### TGA studies

**Fig. 3:**
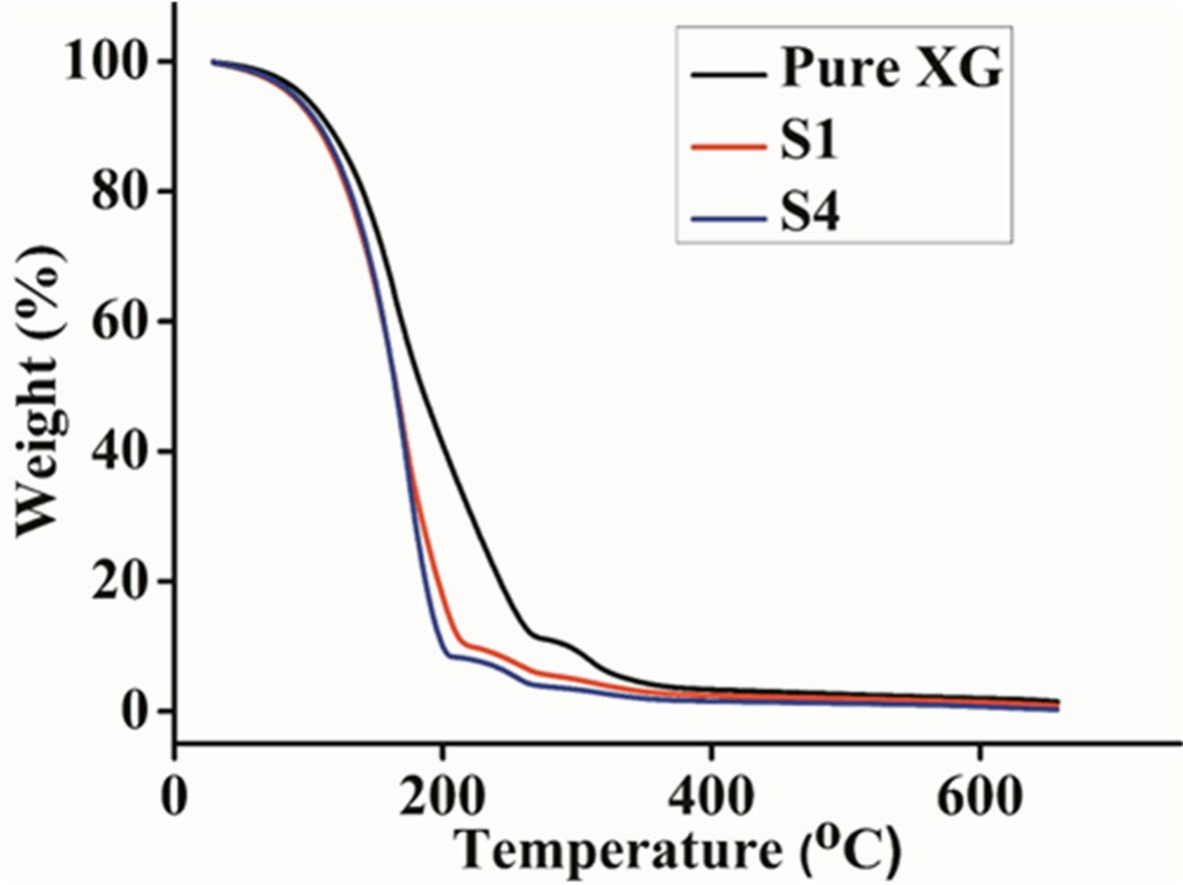
Thermal gravimetric analysis of different gel polymer electrolyte samples.

The decomposition patterns and thermal stabilities of polymers and polysaccharide-based products can be studied accurately via thermal analysis. TGA was performed for the pure XG, S1, and S4 samples (Fig. 3). The relatively small quantity of moisture present in the sample is what caused the initial weight loss in pure XG, which started at approximately 110 °C. The second degradation phase begins at 282 °C and extends until 375 °C due to polymer breakdown. In the case of the plasticized sample containing CQDs, the slight moisture content of the sample caused the initial weight loss, which started at 105 °C. The polymer breakdown begins in the second degradation area at 213 °C and continues until 319 °C. The temperature at which the plasticized NaClO_4_ doped system began to degrade decreased as the salt content increased (S4). For sample S4, constant weight loss begins at 100 °C following the first weight loss caused by the presence of some moisture. Based on the TGA data, the thermal stability of the GPE system was slightly affected by plasticizing and salt doping compared with that of pure XG. This contributes to the conclusion drawn from the FTIR data, which suggests that the polymer and doped salt interact in a certain manner.

Pure XG, S1, and S4 gels exhibit excellent thermal stability and are thermally stable below 100 °C, as evidenced by the thermogram of the thermogravimetric analyses (TGAs) of XG gels, as shown in (Fig. 3). Compared with the pure XG gel, the XG GPE has lower thermal stability because of the inclusion of CQDs. The thermal stability decreased more after the addition of salt. Supercapacitor application behaves effectively at 100 °C. Up to 20% of the total weight reduction with our approach appears to be stable. The relative thermal stability of the XG GPE may be caused by the interaction between the polymer and CQD, which may reduce the crystallinity of the polymer^52^.

### SEM studies

**Fig. 4:**
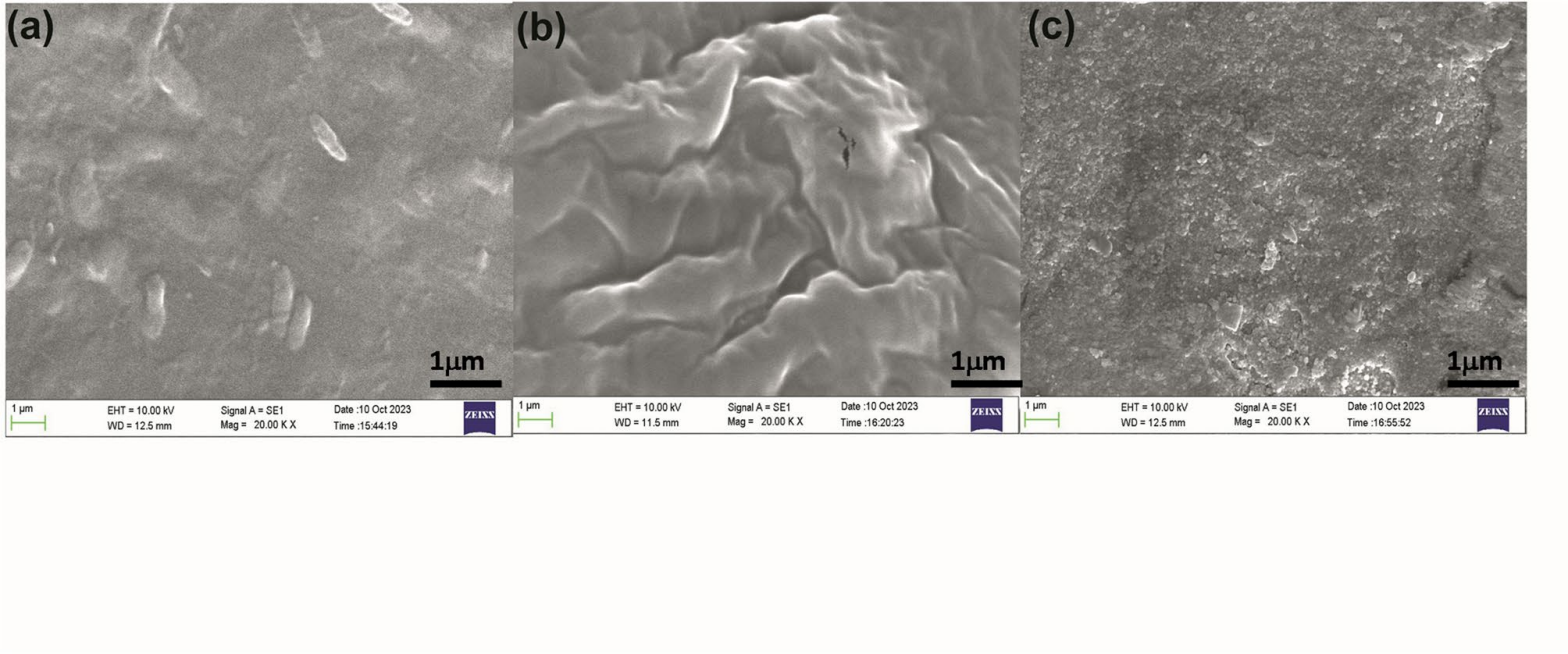
Scanning electron microscope (SEM) images of gel polymer electrolyte samples (**a**) pure XG, (**b**) S1, and (**c**) S4.

Figure 4 (a, b, and c) shows SEM images of the pure XG, S1, and S4 GPE samples. SEM studies revealed crucial insights into the surface morphology of the gel polymer samples. The pristine xanthan gum gel polymer displays a uniform and smooth surface containing few globular structures, suggesting a homogenous composition. In contrast, sample S1, enriched with CQDs, exhibited distinctive planar wave-like features commonly observed in gel polymers. Furthermore, with the addition of NaClO_4_ salt, the wave-like morphology is disrupted and converted to a rougher surface texture, indicating the effective dispersion of the salt throughout the polymer matrix. This dispersion likely contributes to the minimal impact of temperature observed in TGA studies when doped with salt or a plasticizer. In particular, the absence of spherulite formation suggests the amorphous nature of the polymer blend^53^ with successful dissociation of the sodium salt within the polymer matrix, facilitating strong interactions between the salt, plasticizer, and polymer components. Overall, SEM analysis provides valuable visual evidence of the structural modifications induced by various additives, shedding light on the interactions between different components in the gel polymer system.

### TEM studies

**Fig. 5:**
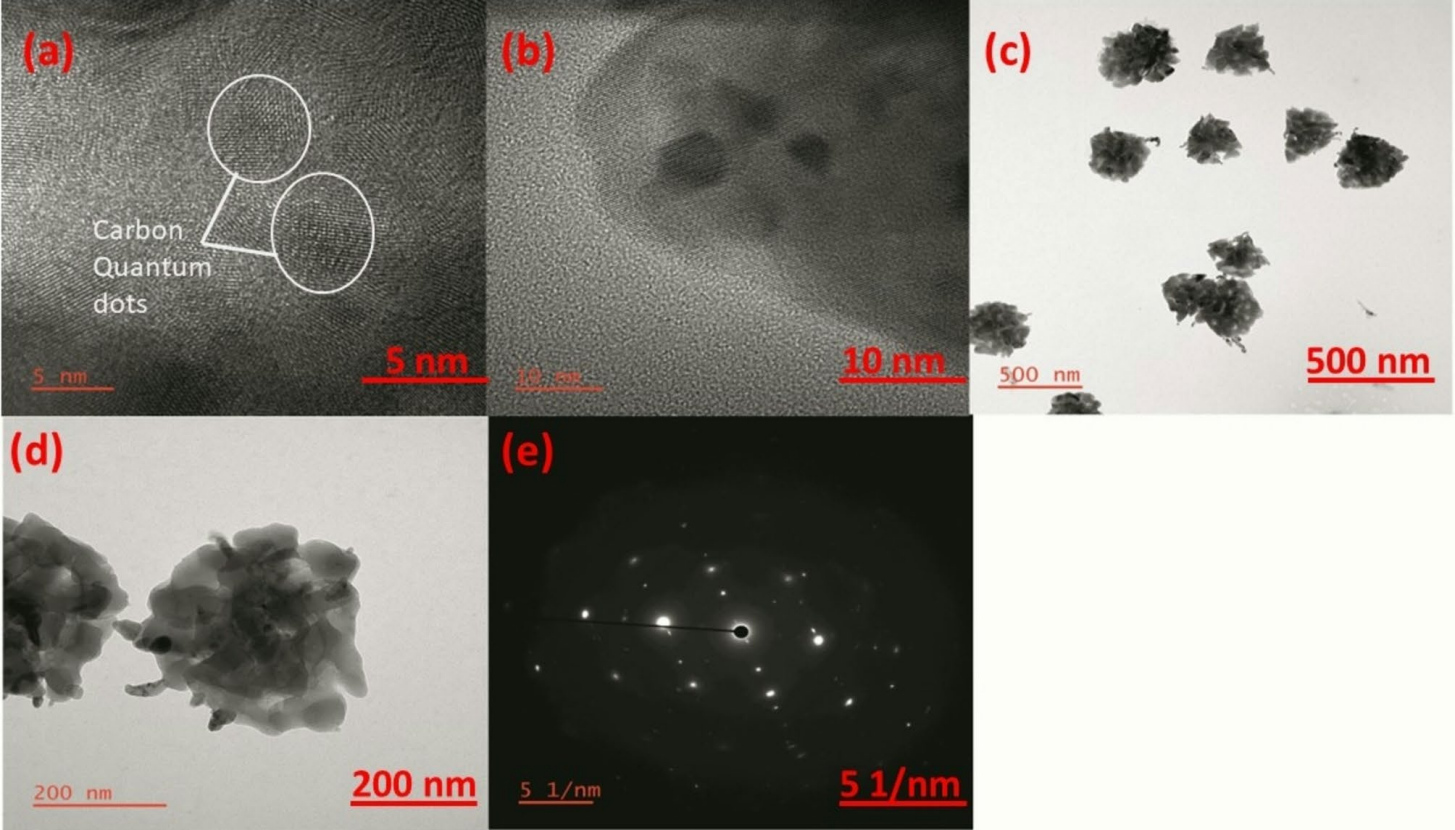
(**a**–**d**) Transmission electron microscopy (TEM) images of the CQD sample. (**e**) Selected area electron diffraction (SAED) pattern of the CQD sample.

Transmission electron microscopy (TEM) studies revealed significant insights into the structural properties and functional enhancements of the xanthan gum-based electrolyte matrix. Initially, the xanthan gum strands appeared elongated and broken down into clusters resembling a puffer ball form within the observed matrix. Furthermore, in this puffer ball, carbon quantum dots of approximately 5 nm are embedded, as depicted in Fig. 5 (a, b, c, d). These observations suggest that while the pure xanthan gum matrix has poor structural integrity, the inclusion of puffer balls of approximately 200 nm significantly enhances its molecular bonding and overall strength. The embedded carbon quantum dots within the puffer balls play a vital role in enhancing the ionic movements during the charge-discharge processes, thereby increasing the electrochemical performance of the matrix. This incorporation approach emphasizes the potential of modifying xanthan gum-based electrolytes for enhancing structural and functional properties in various applications. In our study, the selected area electron diffraction (SAED) pattern of carbon quantum dots synthesized with xanthan gum as a precursor exhibited a distinct hexagonal arrangement, as shown in Fig. 5 (e). This observation suggests that the carbon dots adopted a hexagonal crystalline structure. This structure is characteristic of certain carbon-based materials, such as graphite^54^, which also shows a hexagonal SAED pattern due to its layered arrangement of carbon atoms.

The formation of this hexagonal structure can be attributed to the templating effect of xanthan gum, a polysaccharide known for its helical conformation in aqueous solutions. During synthesis, xanthan gum likely facilitates a specific spatial arrangement of carbon atoms, promoting the formation of hexagonal crystalline domains.

### XRD studies

**Fig. 6:**
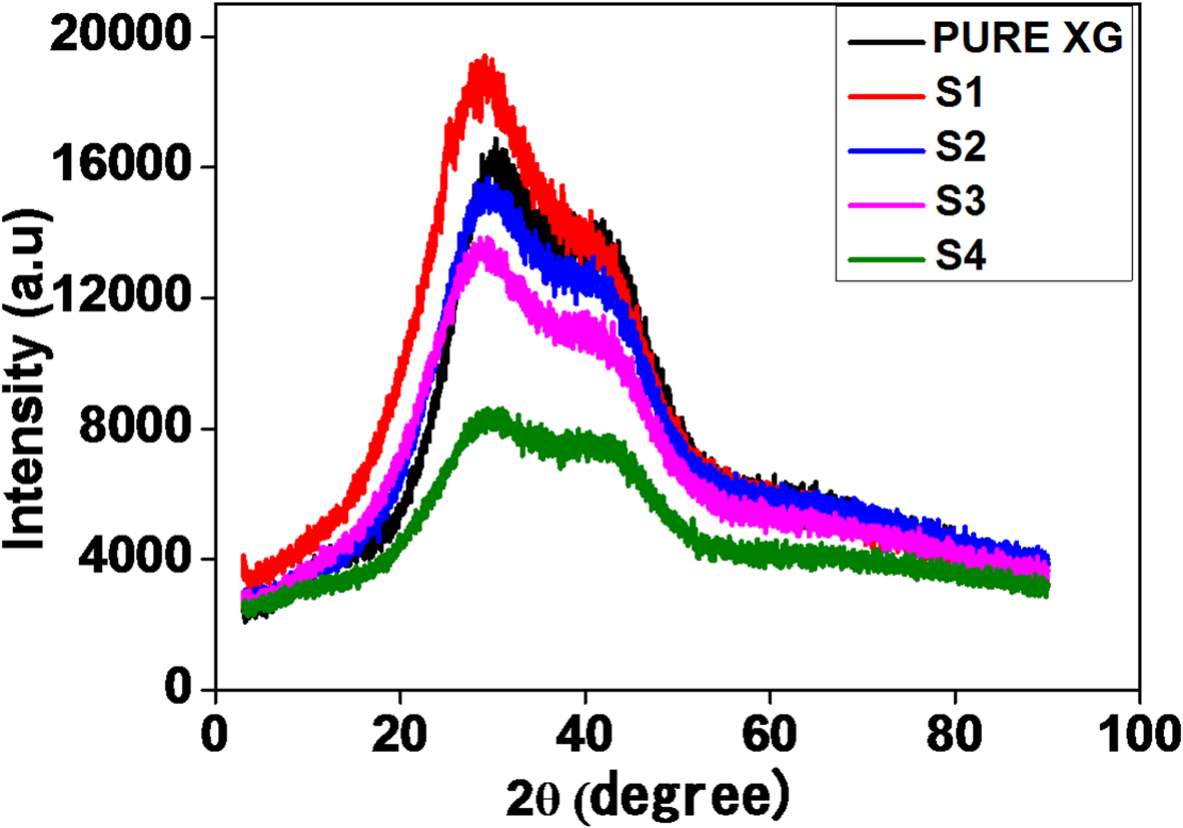
X-ray diffraction (XRD) patterns of different gel polymer electrolyte samples.

Comparative XRD patterns were recorded at room temperature for the pure XG and S1–S4 samples, as illustrated in Fig. 6. The X-ray diffraction (XRD) pattern of XG exhibited a peak in the diffraction angle range of 28° to 30° and a broader peak at approximately 40°. The incorporation of carbon quantum dots (CQDs) increases the intensity of the crystalline peak, with a slight shift in the diffraction angle. Upon further addition of sodium salt, the broad peak at approximately 30° indicates that the gel polymer electrolyte (GPE) primarily comprises an amorphous phase^55^. Doping with 0.01 g of salt results in a slight reduction in the intensity of the crystalline peak at 30°, whereas higher salt concentrations lead to peak broadening. This broadening and disappearance of the crystalline peaks suggest a reduction in crystallinity, thereby lowering the energy barrier for ion conduction^55,56^. With the addition of 0.03 g of salt, a significantly broadened peak is observed, indicating that the sample is the most amorphous. According to Hodge et al..‘s criterion, the reduction in peak intensity and peak broadening signifies a predominant amorphous state within the GPE^57,58^. Analysis of the XRD pattern allows the prediction of the conductivity trend in the XG-GPE, as conductivity is largely dependent on the transport of mobile carriers, which occurs more readily in the amorphous region. Consequently, XG-GPE with 0.03 g of salt has the highest conductivity^53^.

### Supercapacitor studies

#### Cyclic voltammetry of the supercapacitors

**Fig. 7:**
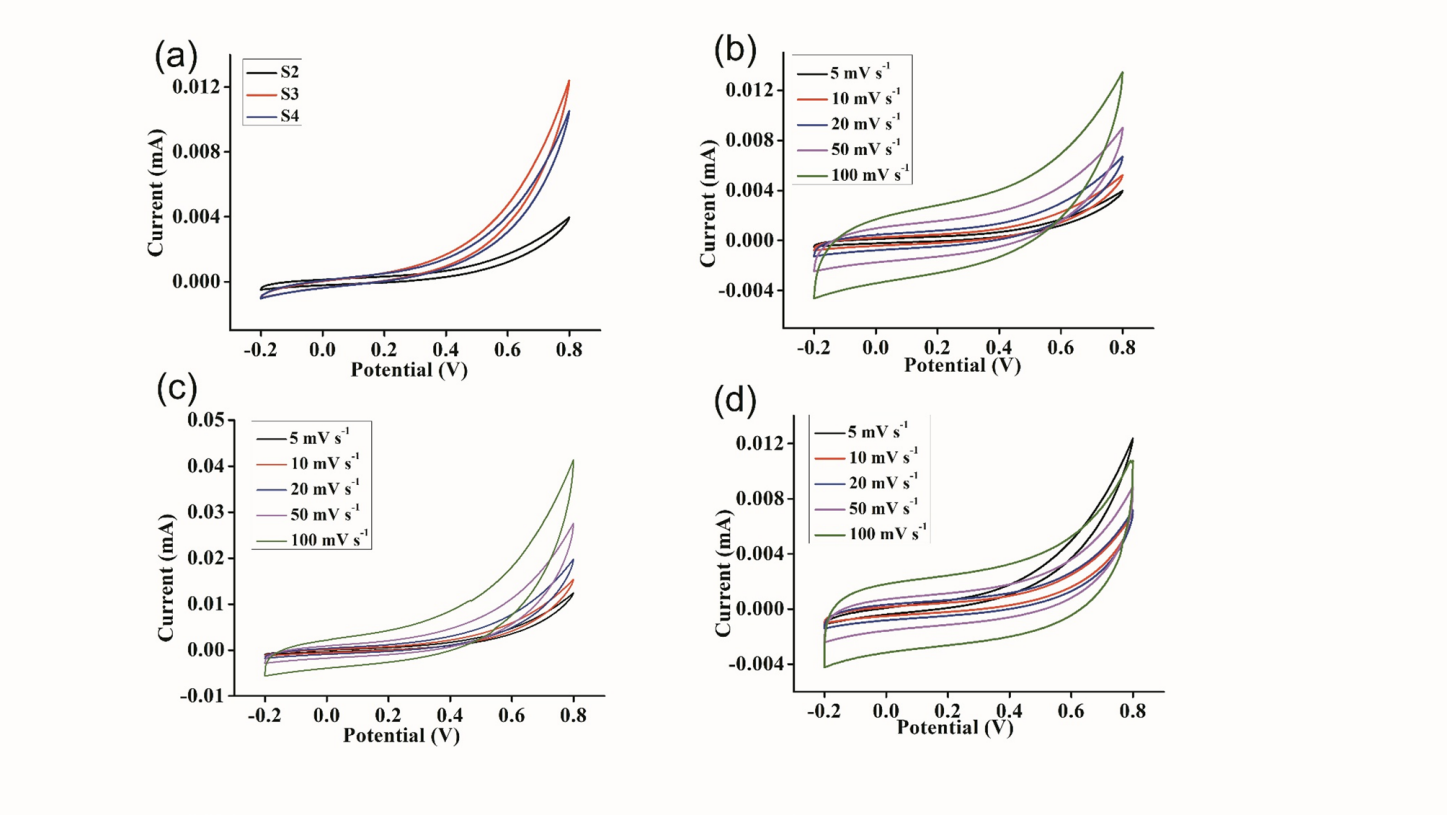
Electrochemical performance of carbon-carbon symmetric electrodes (**a**) CV curves of different GPE samples at a scan rate of 5 mV s^− 1^. (**b**) CV curves of S2 at different scan rates, (**c**) CV curves of S3 at different scan rates, and (**d**) CV curves of S4 at different scan rates.

**Fig. 8:**
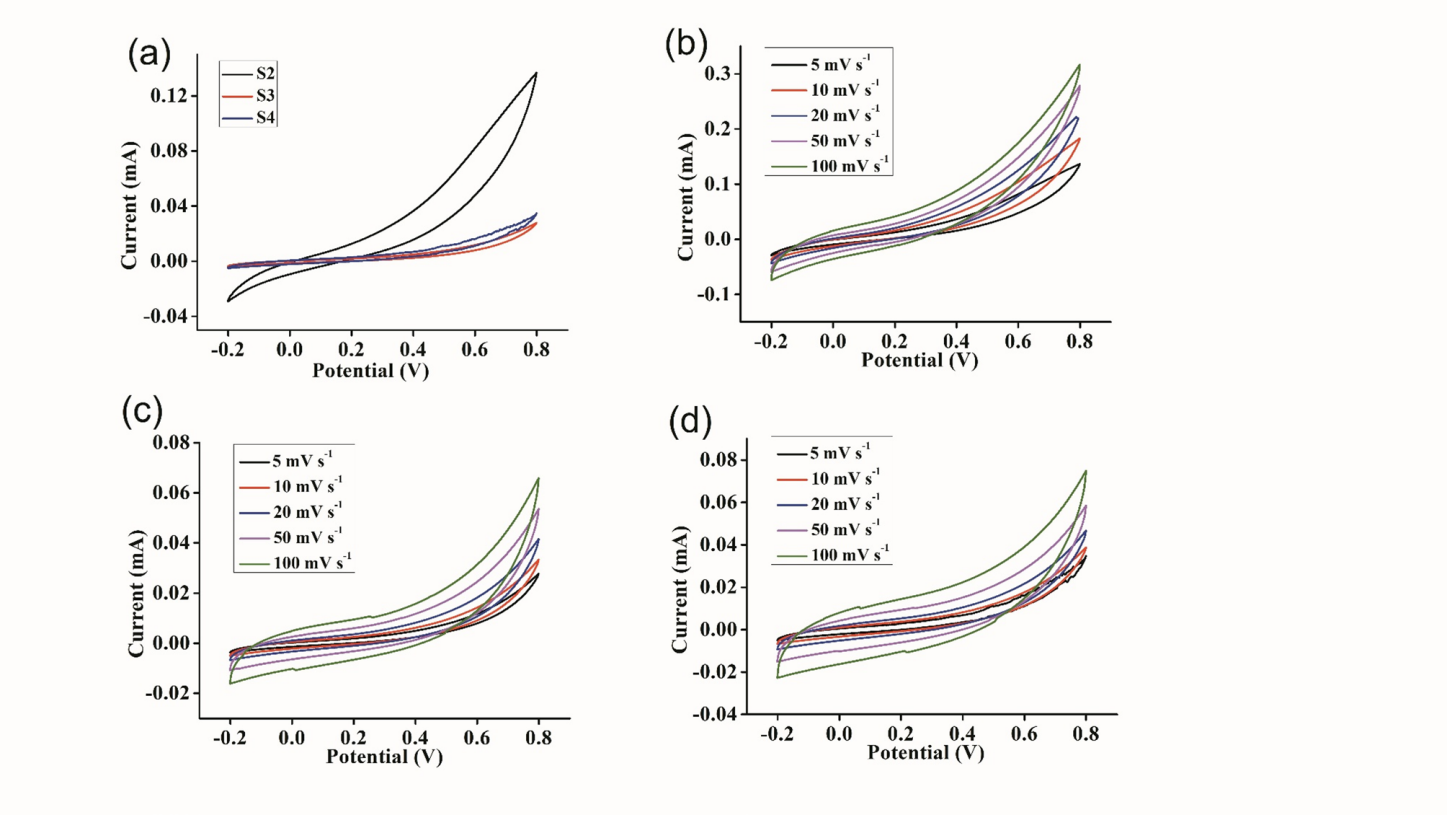
Electrochemical performance of graphene-graphene symmetric electrodes. (**a**) CV curves of different GPE samples at a scan rate of 5 mV s^− 1^. (**b**) CV curves of S2 at different scan rates, (**c**) CV curves of S3 at different scan rates, and (**d**) CV curves of S4 at different scan rates.

The Cyclic voltammetry (CV) responses of the activated carbon and graphene-coated stainless steel containing GPEs at various sweep rates at 303 K are shown in Figs. 7 and 8. The calculations were done using the equation from the literature^59^. The specific capacitance was calculated using Eq. (1).

1 $$\:C=\frac{A}{2mKV}\:$$

Where A is the integrated area of the CV curve, m is mass in g, K is the scan rate in mV s^− 1^ and V is the potential in volts. A maximum specific capacitance of 92 F g^− 1^ at a scan rate of 5 mV s^− 1^ was obtained for a supercapacitor containing an S4 sample coated with AC. The specific capacitance of the supercapacitor coated with graphene was 69 F g^− 1^, at a scan rate of 5 mV s^− 1^ for sample S4. The data reveal that C decreases as the scan rate increases. At lower scan rates, ions have sufficient time to penetrate the pores of the activated carbon materials. Conversely, at higher scan rates, ions tend to accumulate on the external surface of the carbon materials. Consequently, the specific capacitance decreases as the scan rate increases^60^. However, in the AC-coated supercapacitor, the specific capacitance was greater than that in the graphene-coated system. Because of the distortion caused by the solution and gel resistance in the current response at the switching potential, the shapes of the cyclic voltammograms are modified. Diffusion or migration barriers in the electrolyte that prevent ions from reaching the electrode surface may cause distortion^61^.

#### Electrochemical impedance spectroscopy of supercapacitors

**Fig. 9:**
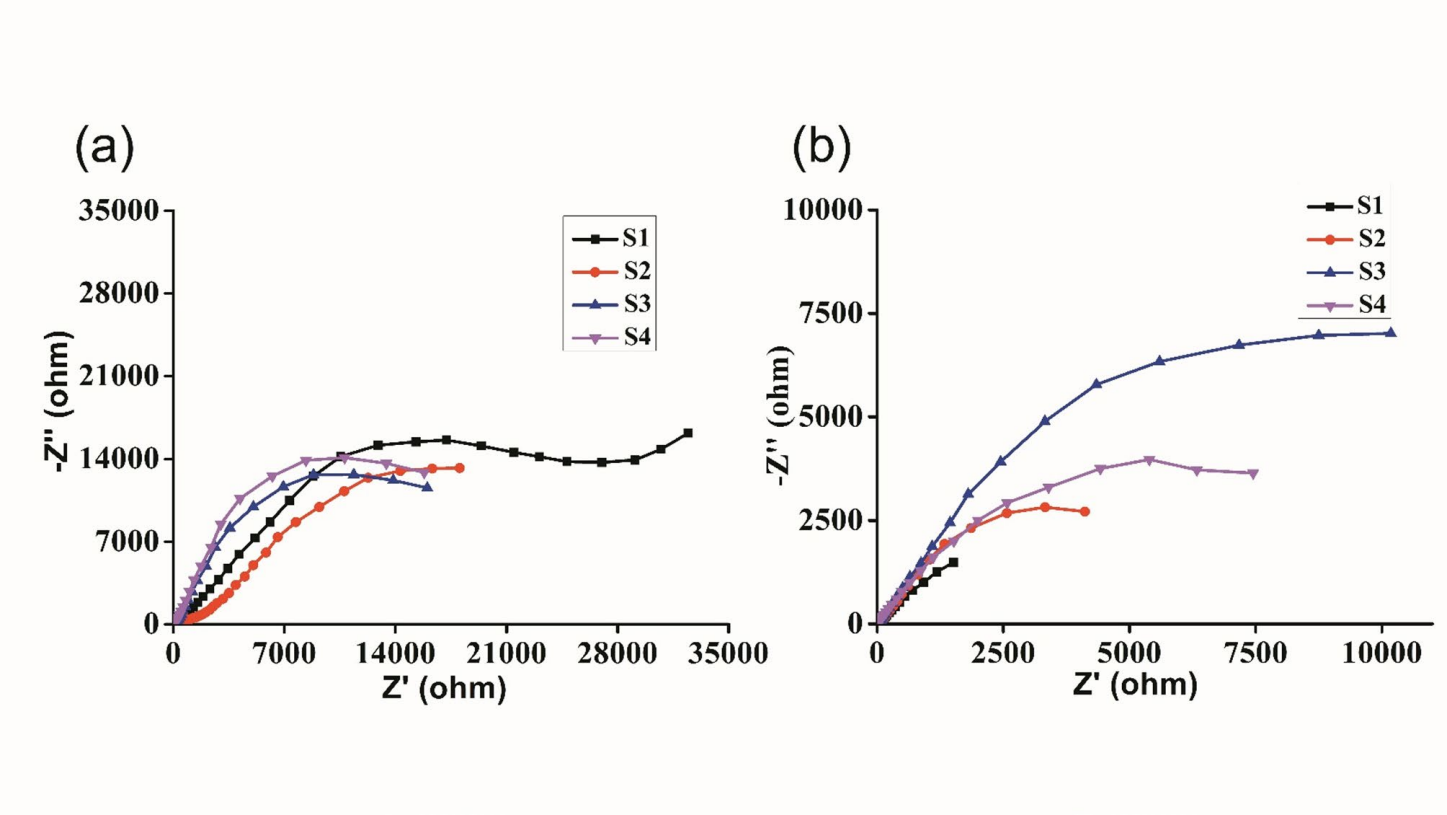
(**a**) Nyquist impedance plots for the AC-coated supercapacitor using the GPE and (**b**) Nyquist impedance plots for the graphene-coated supercapacitor using the GPE.

Figure 9 shows the Nyquist plot of the supercapacitor containing linear regions. The electrochemical impedance spectroscopy was performed at a frequency range from 1 MHz to 100 mHz. The true capacitive behavior of the supercapacitor device prepared from the GPEs is that lower frequencies result in a nearly parallel vertical zone to the imaginary axis, indicating that protons are easily accessible through AC micropores to create a double layer. However, the observed lower impedance of S4 is lower in the higher frequency region than that of S1, S2, and S3, inferring good ionic diffusion behavior of S4. The occurrence of crystalline nonconducting areas in the polymer electrolyte system and the frequency-dependent ohmic resistance caused by uneven double-layer charging could be the causes of this pattern^65^. The presence of any semicircle in the high-frequency region of the Nyquist plot indicates high charge transfer resistance.

#### Galvanostatic charge-discharge (GCD) characteristics of supercapacitors

**Fig. 10:**
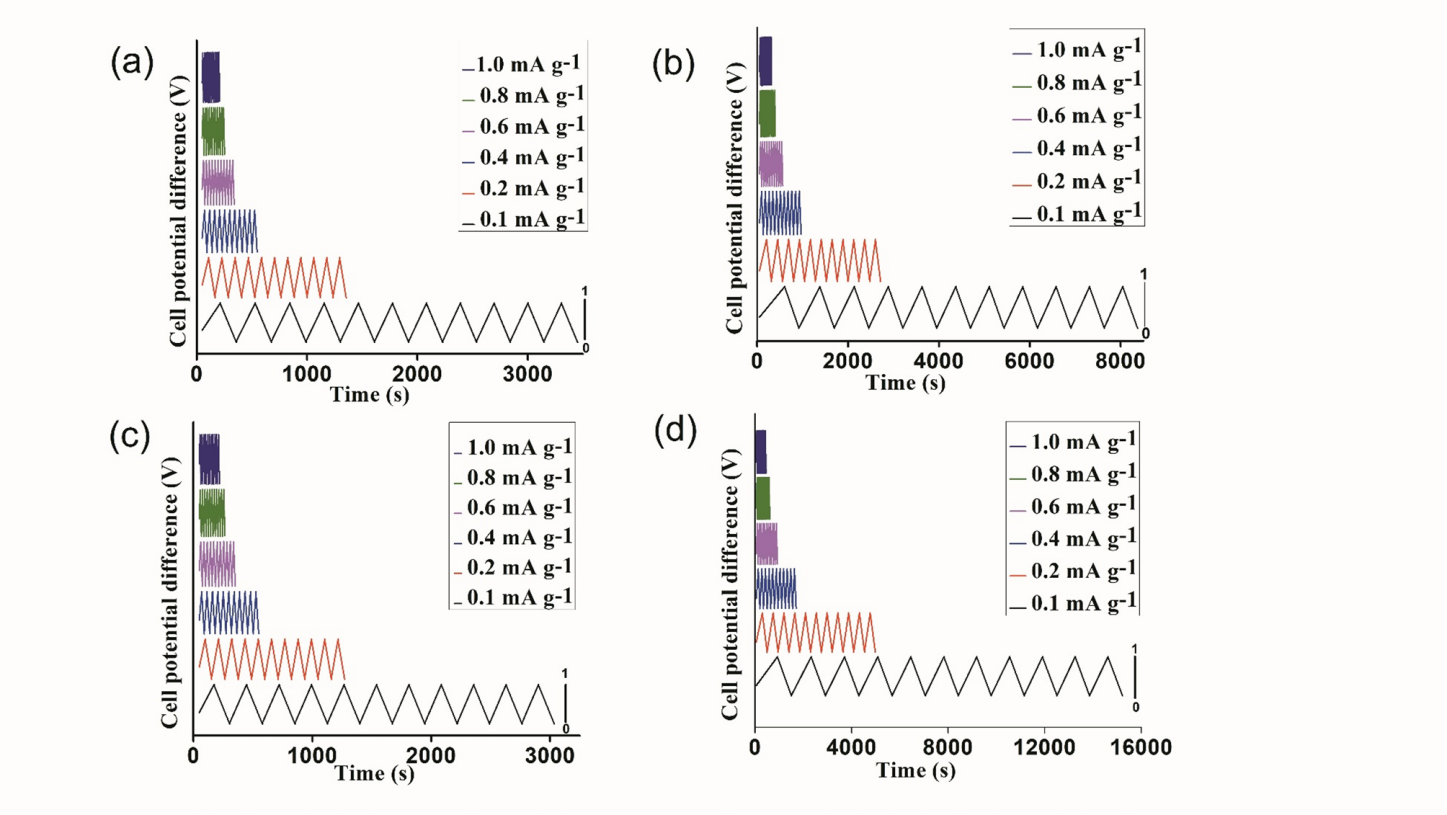
GCD curves of carbon coated supercapacitor at different current densities with GPE samples (**a**) S3, (**c**) S4. GCD curves of graphene-coated supercapacitor at different current densities with GPE samples (**b**) S3, (**d**) S4.

GCD measurements were performed for both AC and the graphene-coated supercapacitor doped with NaClO_4_ to investigate how the performance of the constant current charge-discharge characteristics is affected by the ionic conductivity. Figure 10 displays the supercapacitor charge-discharge behavior as determined by the galvanostatic method by varying the current density to 0.1 mA g^− 1^, 0.2 mA g^− 1^, 0.4 mA g^− 1^, 0.6 mA g^− 1^, 0.8 mA g^− 1^ and 1 mA g^− 1^ between potential windows of -0.2–0.8 V. In Galvanostatic Charge-Discharge (GCD) curves, the charging and discharging durations decrease as current density increases because electrolyte ions’ surface adsorption and diffusion into the electrode’s active material slow down^62–64^. At lower current densities, the slower charge accumulation allows electrolyte ions to diffuse more effectively, enhancing their access to active sites on the electrode. This facilitates a more complete insertion/extraction process, improving specific capacity^62–65^. The specific capacitance (C_s_, F g^− 1^) of the supercapacitor was evaluated from the literature^66^ via the following Eq. (2).

2 $$\:Cs=2\frac{I\times\:\varDelta\:t}{\varDelta\:V\times\:m}$$

where C_s_ represents the specific capacitance of the device in F g^− 1^, △V (V) denotes the voltage change after a full charge or discharge, m (g) is the weight of the active material (including the binder and active material), △t (S) is the discharge time, and Ir drop (V) is the electrical potential difference between the two ends of a conducting phase during charging-discharging. The fabricated device showed a good specific capacitance of 75 F g^− 1^ for the S4 sample with graphene coating at the current density of 1 mA g^− 1^. Furthermore, the specific capacitance values for the devices fabricated with different GPE samples are presented in Table 2.

**Table 2:** Values of the specific capacitance C_s_, at a constant current of 1 mA g^− 1^ were obtained from the charge-discharge characteristics.

Samples	XG-S4/AC	XG-S4/GC	XG-S3/AC	XG-S3/GC
C_s_ (F g^− 1^)	40.04	75	20.02	40

Further, the energy density (E_D_) and power density (P_D_) was evaluated from literature^67^ using Eqs. (3) and (4) as follows:

3 $$\:{E}_{D}=\frac{1}{2}Cs{(\varDelta\:V)}^{2}\times\:\frac{1000}{3600}$$

4 $$\:{P}_{D}=\frac{{E}_{D}\times\:3600}{\varDelta\:t}$$

where E_D_ (W h kg^− 1^) is the energy density, C_s_ (F g^− 1^) is the specific capacitance, DV(volts) is the operating voltage. P_D_ (W kg^− 1^) is the energy density and Dt (s) is the time window during the potential range.

Table 3 presents a comparison of the best performance of XG-S4 in this study with other reported works that used different active materials as the active electrode material. Owing to the strong ionic conductivity and flexibility of the GPE-S4 electrolyte, which provides good contact between the electrode and electrolyte, the supercapacitor containing it has good power density in comparison with other materials reported in the literature^68^.

**Table 3:** Comparison of the electrochemical parameters of supercapacitor cells fabricated with different gel polymer electrolytes.

Gel polymer electrolytes (GPEs)	C_s_ (F g^− 1^)	Voltage (V)	E (W h kg^− 1^)	*P* (W kg^− 1^)	Refs.
PILTFSI/PYR14FSI	150	3.5	36	230	^69^
Agar/Na_2_SO_4_	196.4	1.8	22.1	450	^70^
Agarose/NaCl	286.9	0.8	–	–	^71^
Guar gum/LiClO_4_	186	1.0	–	–	^27^
Soybean straw/KOH	380.5	–	8.95	25	^72^
Pecan shell/KOH	447	–	15.5	62	^73^
GPE/activated charcoal	157	–	–	–	^74^
GO-PGE/FCNT	83.3	1.0	–	–	^75^
PGPE/AC	64.92	1.2	13.26	226	^76^
XG-S4/GC	75	1.0	10.40	490	Current work
XG-S4/AC	40	1.0	5.55	660	Current work

##### Mechanism

**Fig. 11:**
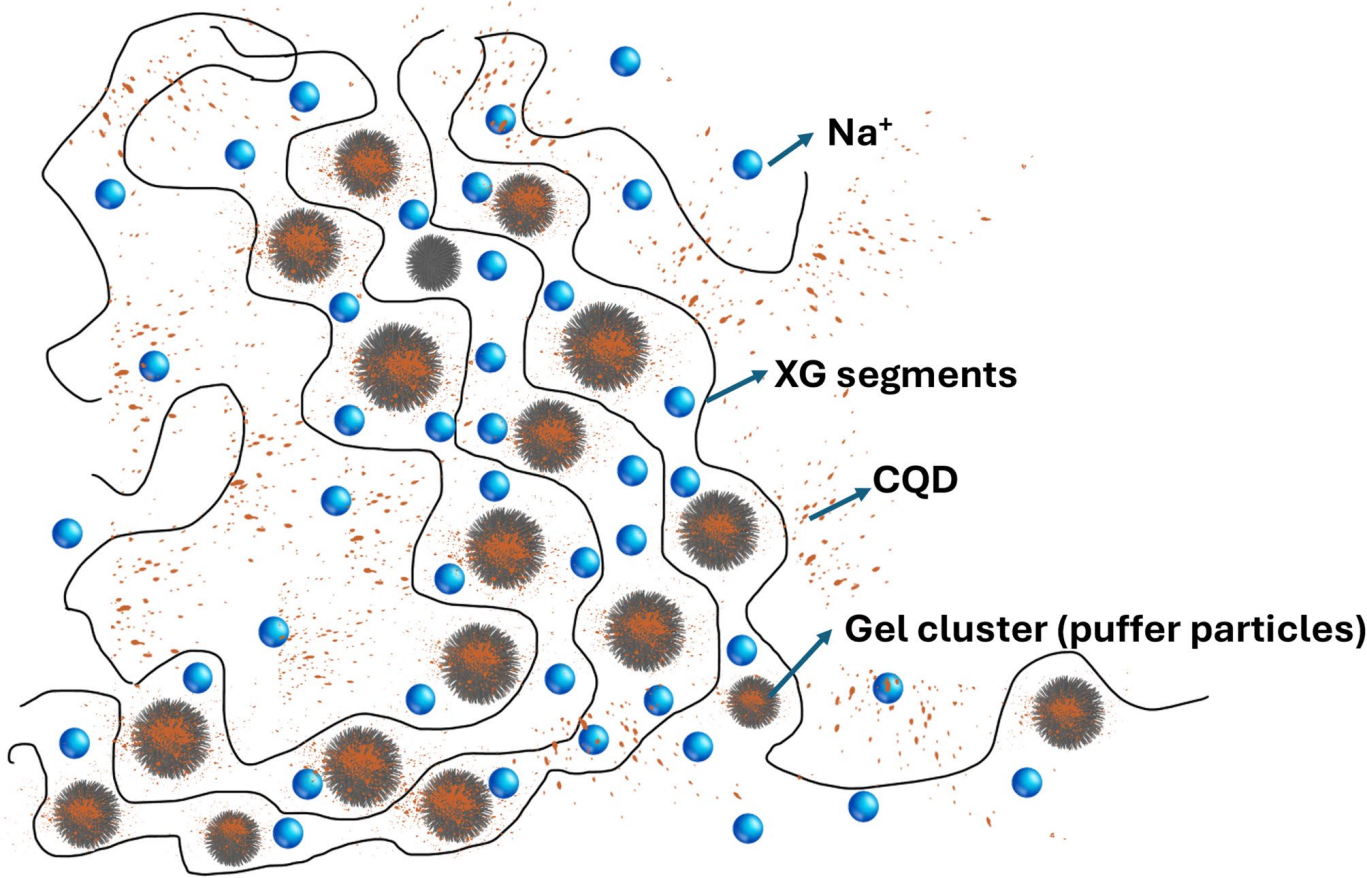
Probable interactions in XG/CQD/Na salt polymer electrolytes.

In the gel polymer electrolyte of a supercapacitor, the probable interaction between the xanthan gum, carbon quantum dot and salt as shown in Fig. 11. XG serves as the structural matrix, providing a robust and flexible network that maintains mechanical stability and flexibility while creating a porous structure for ion movement. Glycerol, which acts as a plasticizer, intercalates between xanthan gum polymer chains, increasing the free volume, increasing pliability, and improving the ionic conductivity by facilitating easier ion transport. Sodium perchlorate dissociates into sodium (Na⁺) and perchlorate (ClO₄⁻) ions, which serve as charge carriers; their high concentration reduces resistance and enhances supercapacitor performance. Puffer ball-like structures facilitate interactions and effectively provide a channel for sodium ions within the segments of xanthan gum. Carbon quantum dots (CQDs) are incorporated to further improve conductivity and electrochemical properties because of their excellent electrical conductivity and high surface area. At the molecular level, the oxygen-containing functional groups (such as hydroxyl and carboxyl) on the surface of CQDs can form hydrogen bonds and electrostatic interactions with the hydroxyl and carboxyl groups present in the XG backbone. These interactions not only enhance the dispersion of CQDs within the biopolymer matrix but also lead to partial charge transfer between the CQDs and XG. CQDs enhance electrolyte‒electrode interactions, provide additional active sites for ion adsorption/desorption, and help in the homogeneous distribution of ions. Activated carbon (AC) in supercapacitor electrodes enhances performance by establishing a porous network, facilitating efficient diffusion of electrolyte ions. This structure increases the surface area of the electrode for enhanced ion adsorption and desorption during charge and discharge cycles, thereby increasing the energy storage capacity. The synergistic interactions among xanthan gum, glycerol, sodium perchlorate, and CQDs result in high ionic conductivity, facilitating efficient charge/discharge cycles and leading to improved power density and overall performance of the supercapacitor.

## Conclusion

A supercapacitor with an activated carbon/graphene electrode and an eco-friendly xanthan gum-based gel polymer electrolyte was successfully fabricated, showcasing the promise of sustainable materials in energy storage. The addition of carbon quantum dots (CQDs) to the GPE yielded a unique wave-like surface morphology and increased the amorphous content, as confirmed by SEM and XRD analyses. A fabricated carbon-carbon supercapacitor demonstrated a specific capacitance of 92 F g^− 1^ with a maximum energy density of 10.40 Wh kg⁻¹ and a power density of 0.66 kW kg⁻¹. The supercapacitor showed good power density, energy density, and stability during charge-discharge cycles. This study highlights the potential of biodegradable GPEs, which incorporate sodium perchlorate (NaClO_4_) as a dopant, glycerol as a plasticizer, and CQDs as precursors. Thermal analyses revealed the influence of NaClO_4_ and the CQDs on the thermal stability of the GPE. Electrochemical evaluations revealed promising energy density and specific capacitance values, whereas galvanostatic charge-discharge studies highlighted the considerable influence of current density on overall performance. The results demonstrate that the device capacitance is significantly impacted by the ionic conductivity of the GPE, the interaction of the electrolyte with salt ions, and the properties of the electrode material. This research introduces the innovative integration of carbon quantum dots (CQDs) into gel polymer matrices, highlighting their potential to revolutionize materials science through enhanced optical, electrical, and mechanical properties. While promising, this field is in its early stages, requiring further exploration of synthesis techniques, CQD‒polymer interactions, and scalability for commercial applications. Future research should focus on tailoring functionalities for applications in energy storage, sensors, bioimaging, and environmental remediation while addressing stability, environmental impact, and recyclability to ensure sustainability.

## Electronic supplementary material

Below is the link to the electronic supplementary material.

Supplementary Material 1

## Data Availability

All data generated or analysed during this study are included in this published article and its supplementary information files.

## Declarations

### Generative AI and AI-assisted technologies in the writing process

During the preparation of this work, the author(s) utilized ChatGPT and AGE to facilitate idea generation, refine language, and ensure clarity of expression. Following the use of these tools, the author(s) thoroughly reviewed and edited the content to align with their research objectives and academic standards. The author(s) take full responsibility for the accuracy and integrity of the content presented in this publication.
